# Modulatory Effect of *Pyrus pyrifolia* Fruit and its Phenolics on Key Enzymes against Metabolic Syndrome: Bioassay-Guided Approach, HPLC Analysis, and *In Silico* Study

**DOI:** 10.1007/s11130-023-01069-3

**Published:** 2023-05-23

**Authors:** Nariman E. Mahdy, Passent M. Abdel-Baki, Ahmed A. El-Rashedy, Rana M. Ibrahim

**Affiliations:** 1 Pharmacognosy Department, Faculty of Pharmacy, Cairo University, Kasr-El-Ainy Street, Cairo, 11562 Egypt; 2 Natural and Microbial Products Department, National Research Center (NRC), Dokki, Giza, 12622 Egypt

**Keywords:** *Pyrus pyrifolia*, HPLC, Phenolics, Antioxidant activity, Metabolic syndrome, Molecular dynamic.

## Abstract

This study aims to isolate the active constituents of *Pyrus pyrifolia* Nakai fruits using a bioassay-guided fractionation approach, test their activity in vitro against key enzymes for metabolic disorders, and support it with molecular docking simulations. The antioxidant potential of the methanolic extract (ME), its polar (PF), and non-polar fractions (NPF), along with the inhibitory activity against *α*-glucosidase, *α*-amylase, lipase, angiotensin I converting enzyme (ACE), renin, inducible nitric oxide synthase (iNOS), and xanthine oxidase (XO) were assessed. The PF exhibited the highest antioxidant and enzyme inhibitory activity. Purification of PF yielded rutin, isoquercitrin, isorhamnetin-3-*O*-*β*-D-glucoside, chlorogenic acid, quercetin, and cinnamic acid. HPLC-UV analysis of the PF allowed for the quantification of 15 phenolic compounds, including the isolated compounds. Cinnamic acid was the most powerful antioxidant in all assays and potent enzyme inhibitor against the tested enzymes (*α*-glucosidase, *α*-amylase, lipase, ACE, renin, iNOS, and XO). Additionally, it showed high affinity to target *α*-glucosidase and ACE active sites with high docking scores (calculated total binding free energy (ΔGbind) -23.11 kcal/mol and − 20.03 kcal/mol, respectively]. A 20-ns molecular dynamics simulation using MM-GBSA analysis revealed a stable conformation and binding patterns in a stimulating environment of cinnamic acid. Interestingly, the isolated compounds’ dynamic investigations including RMSD, RMSF, and Rg demonstrated a stable ligand − protein complex to the active site of iNOS with ΔGbind ranging from − 68.85 kcal/mol to -13.47 kcal/mol. These findings support the notion that *P. pyrifolia* fruit is a functional food with multifactorial therapeutic agents against metabolic syndrome-associated diseases.

**Supplementary Information:**

The online version contains supplementary material available at 10.1007/s11130-023-01069-3.

## Introduction

Metabolic syndrome (MS) is a cluster of cardiometabolic risk factors including hypertension, hyperglycemia, and obesity, as well as pro-oxidant and pro-inflammatory conditions that predispose to chronic diseases (cardiovascular diseases and diabetes) [1]. According to the World Health Organization, MS is regarded as a noncommunicable disease that accounts for 41 million annual fatalities (71% of all deaths worldwide) [2]. Plants are becoming increasingly popular due to their health benefits all over the world. [3]. Fruit consumption has been correlated with a lower incidence of MS [4]. It is also widely accepted that some of these health advantages can be related to the phenolic contents found in edible fruits, which have antioxidant properties [4]. A comprehensive treatment strategy for MS includes radical scavenging activity, as well as inhibition of key enzymes responsible for carbohydrate (*α*-glucosidase and *α*-amylase), and lipid metabolism (pancreatic lipase), hypertension [angiotensin I converting enzymes (ACE) and renin], oxidative stress [xanthine oxidase (XO)], and pro-inflammatory conditions [inducible nitric oxide synthase (iNOS)] [1].

Pears (*Pyrus* spp.) are widely grown around the world. They are popular for their sweetness, freshness, distinctive scent, and mild aroma [5]. There are two main types of pears: “European” and “Asian” pears. While Asian pears are often rich in water and phenolics with lower sugar and starch contents, European pears have larger calorie and sugar contents. Thus, Asian pears are known as a nutritionally healthy fruit [5]. *P. pyrifolia* Nakai (Asian pear) fruits are cultivated mainly in Eastern Asia. They are usually consumed fresh and/or processed as juices, jams, and jellies [6]. In addition, *P. pyrifolia* exhibited a wide range of biological activities, including anti-inflammatory, antioxidant, antimicrobial, antidiabetic, and cytotoxic potentials, owing to the presence of various active constituents such as chlorogenic acid, gentisic acid, arbutin, catechin, epicatechin, quercetin, rutin, and leupirol [7]. Lifestyle modifications, depending on a healthy diet, have been regarded as a first-line treatment for MS. This prompted us to investigate the phytochemical profile of *P. pyrifolia* fruits’ extract and fractions, as well as their potential antioxidant activity, using 2,2’-azino-di(3-ethylbenzthiazoline-6-sulfonic acid (ABTS), ferric reducing antioxidant power (FRAP), and oxygen radical absorbance capacity (ORAC) assays. Furthermore, the ability of the fruit extract and fractions to inhibit key enzymes involved in MS was tested using in vitro assays. Bioactive compounds were isolated from the most promising fraction and investigated for their respective biological potential against MS-related enzymes by adopting a bioassay-guided fractionation process. Using high-performance liquid chromatography with ultraviolet (HPLC-UV) analysis, the isolated compounds and other phenolic constituents were quantified in the active fraction. Additionally, the binding affinities of the isolated compounds to the active sites of *α*-glucosidase, ACE, and iNOS were studied using molecular docking simulations to provide a mechanistic perception of their biological and therapeutic potentials holistically for the first time. These results may contribute to the incorporation of Asian pear extract or its polyphenols as part of the strategy against diabetes-, obesity-, and hypertension-related metabolic disorders.

## Materials and Methods

The detailed procedures are described in the Supplementary online resource.

### Plant Material

*Pyrus pyrifolia* Nakai ripe fruits were collected in August (2022) from Makar Farms, Giza Governorate, Egypt. The plant material was authenticated and verified by Botany specialist, Dr. Mohamed El-Gibali, former researcher of Botany, Department of Botany, National Research Center (NRC). A voucher specimen was deposited in Pharmacognosy Department Herbarium, Cairo University (registration no. 9.09.2022).

### Preparation of the Methanolic Extract (ME) and its Fractionation

Fresh pears (3 kg) were freeze-dried, homogenized, and extracted with methanol (7 L x 4) till exhaustion at room temperature, and the combined extracts were evaporated in vacuo in a rotary evaporator (50 °C). The crude extract (180 g) was subjected to liquid-liquid fractionation with methylene chloride (1 L x 4), followed by *n*-butanol saturated with water (1 L x 4), and both were concentrated to give non-polar (NPF) (60 g) and polar fraction (PF) (120 g), respectively.

### Isolation of the Major Compounds from the Polar Fraction (PF)

The PF (15 g) was chromatographed over a polyamide column (5 D x 25 L cm) eluting with 0–100% methanol in water to afford 5 fractions (I-V). Fraction II (1.7 g) was chromatographed on a Sephadex LH-20 column (3 D X 30 L cm) using methanol: water (1:1 v/v) for elution, yielding two subfractions; II-A and II-B. Subfraction II-A (0.57 g) was purified on RP-18 column (1 D x 10 L cm) using water-methanol mixtures to yield compound **P1** (yellow crystals, 115 mg). Fraction III (2.4 g) was chromatographed on a Sephadex LH-20 column (3 D X 30 L cm) using methanol: water (1:1 v/v) for elution, yielding three main subfractions; III-A, III-B, and III-C. Subfractions III-A (0.85 g), III-B (0.75 g), and III-C (0.70 g), each was separately purified on Sephadex LH-20 column (3 D X 30 L cm) eluted with methanol: water (1:1 v/v) to yield compounds **P2** (yellow crystals, 89 mg), **P3** (yellow crystals, 103 mg) and **P4** (white crystals, 105 mg), respectively. Fraction IV (2.95 g) was loaded onto a Sephadex LH-20 column (3 D X 30 L cm) and eluted with 50% aqueous methanol to give 3 subfractions; IV-A, IV-B, and IV-C. Subfraction IV–C (2.1 g) was chromatographed over a silica gel 60 column, (3 D x 25 L cm, 70–230 mesh), and eluted with methylene chloride: methanol (95: 5 v/v) affording compounds **P5** (yellow crystals, 210 mg) and **P6** (white crystals, 180 mg). The scheme for chromatographic fractionation of PF is represented in Fig. S1.

## Results and Discussion

### Phytochemical Assessment of the ME, NPF, and PF

The ME, NPF, and PF were evaluated regarding their total phenolic (TPC) and flavonoid (TFC) contents (Table S1). Herein, the highest TPC and TFC were found in the PF (20.28 ± 0.17 µg gallic acid equivalent (GAE)/mg dried weight (DW) and 16.39 ± 0.52 µg quercetin equivalent (QE)/mg DW, respectively). The ME showed higher TPC (12.18 ± 0.42 µg GAE/mg DW) than that previously reported in *P. pyrifolia* fruits methanolic extract (9.4 ± 0.06 µg GAE/mg DW) [8].

### Biological Assessment of the ME, NPF, and PF

#### Antioxidant Activity

Oxidative stress is a major cause of the pathogenesis of inflammatory, metabolic, and cardiovascular, diseases [9]. In addition, the observed excessive production of reactive oxygen species in MS has been shown to aggravate hypertension and cardiovascular diseases [10]. To alleviate this pathological condition, exogenous antioxidants are required to provide synergistic activity with the endogenous antioxidant defense system [9]. Assessment of the antioxidant potential of the ME, NPF, and PF was done using three different assay models *viz.;* radical scavenging activity (ABTS), redox potential (FRAP), and ORAC (Table S1). In the three assays, the PF showed the highest antioxidant potential equivalent to 270.97 ± 0.35, 139.11 ± 0.15, and 301.84 ± 2.01 µM Trolox equivalent (TE)/g, representing 94.16, 89.62, and 77.35% antioxidant activity compared to ascorbic acid (reference drug) in ABTS, FRAP, and ORAC, respectively. This powerful antioxidant activity is likely due to the presence of high TPC and TFC. In a previous study, the peels of *P. pyrifolia* showed high ABTS activity that is nearly similar to our results [11]. As far as our literature survey could ascertain, this is the first report regarding the ABTS and ORAC activities of *P. pyrifolia* fruits.

### Inhibitory Activity Against Key Enzymes Related to MS

Inhibition of key enzymes responsible for starch breakdown and glucose absorption (*α*-glucosidase and *α*-amylase) is an effective antidiabetic treatment. Furthermore, inhibition of pancreatic lipase, responsible for dietary lipids breakdown and their intestinal absorption, is regarded as anti-obesity treatment [1]. Concurrent inhibition of renin and ACE activity would effectively regulate the renin-angiotensin system which significantly influences blood pressure, offering an effective treatment option for hypertension [12]. XO inhibition was proven to reduce oxidative stress by lowering uric acid levels [13]. Pro-inflammatory conditions are associated with the etiopathology of MS. This is evidenced by increased nitric oxide (NO) production by the inducible isoform of the enzyme nitric oxide synthase (iNOS). NO has been thought to increase tissue damage because it accelerates the synthesis of reactive nitrogen species. Thus, inhibition of iNOS induction mediated by pro-inflammatory cytokines may improve MS-related alterations [14]. Therefore, inhibiting these enzymatic pathways is regarded as an effective therapeutic approach for illnesses associated with MS. The ME, NPF, and PF inhibitory activity against the enzymes related to MS was evaluated (Table S2). The PF exhibited the highest inhibitory activity followed by ME. The NPF exhibited the least enzyme inhibitory potential. As proven by the effectiveness of the PF with the highest TPC, TFC, and antioxidant potential, this investigation demonstrated a substantial link between the MS-related enzymes’ inhibitory action and the phenolic and flavonoid contents. In general, phenolic compounds have high affinity for proteins through hydrogen and hydrophobic interactions, which allows them to inhibit the enzymes. The interactions that are made possible by the functional groups of the phenolic compounds cause the enzyme to become denaturized and their catalytic activity to be diminished [15].

### Phytochemical Assessment of the Biologically Active Fraction (PF)

Among the fractions studied, the PF was the best candidate for further phytochemical investigation due to its high phenolic and flavonoid contents, as well as its significant antioxidant and enzyme inhibitory activities. Thus, this fraction was subjected to extensive chromatographic fractionation and purification (Fig. S1) to isolate its major active constituents and evaluate their antioxidant and inhibitory activity against the key enzymes related to MS, which is then evidenced by detailed docking studies. Six phenolic compounds (P1-P6) were isolated and identified by their physicochemical characters, UV spectral data, spectroscopic analyses (^1^ H and ^13^ C), and comparison with the available literature (Tables S3 and S4). The identities of the isolated compounds were established as P1: rutin [8], P2: isoquercitrin [16], P3: isorhamnetin-3-*O*-*β*-D-glucopyranoside [17], and P4: chlorogenic acid [17], P5: quercetin [17], and P6: cinnamic acid [18]. The chemical structures of compounds P1-P6 are illustrated in Fig. S2.

### HPLC Analysis of the Biologically Active Fraction (PF)

HPLC analysis revealed that the PF was rich in phenolic compounds. In total, 15 phenolics (9 phenolic acids and 6 flavonoids) were identified and quantified (Table S5) using the available standards. The HPLC chromatogram is shown in Fig. S3. The occurrence of the 6 isolated compounds was confirmed by HPLC analysis. Chlorogenic and cinnamic acids were the major phenolic acids identified with a concentration of 1888.39 ± 0.58 and 998.79 ± 0.32 mg/100 g DW, respectively. Two flavonoid classes were detected: flavonol and flavone. The flavonol class was represented by 6 compounds, the most abundant of which was quercetin (2053.94 ± 0.99 mg/100 g DW), followed by isorhamnetin-3-*O*-glucoside, kaempferol, isoquercitrin, and rutin. Apigenin was the detected flavone. The results were consistent with previous studies that identified chlorogenic acid as the major constituent in *P. pyrifolia* peels [19]. The profile closely resembled that previously reported by Li et al. [20] as both share nearly similar compounds. HPLC-determined compounds, such as phenolic acids, flavonols, and flavones, are extensively reported as potent antioxidants and promising drugs in the prevention and treatment of MS. They are proven to have free radical scavenging activities, antidiabetic, lipid-lowering, antihypertensive, and inhibitory pro-inflammatory actions [21]. This fruit appears to be suitable for the pharmaceutical and functional food industries based on its phenolic composition.

### Antioxidant Activity of the Isolated Compounds (P1-P6)

The antioxidant activity of the isolated compounds was evaluated using ABTS, FRAP, and ORAC (Table S6). All compounds showed powerful antioxidant activity in the 3 assays ranging from 92.91–62.64% compared to ascorbic acid. Cinnamic acid (P6) exhibited the highest antioxidant potential in the three assays, exhibiting 250.28 ± 0.21, 141.26 ± 0.91, and 362.55 ± 1.20 µM TE/g, followed by quercetin representing 244.01 ± 0.29, 139.03 ± 0.65, and 358.95 ± 0.78 µM TE/g, respectively. The high antioxidant capacity of cinnamic and chlorogenic acids is linked to the propenoic side chain that stabilizes the phenoxyl radical by resonance [22]. Regarding flavonoids, their antioxidant activity is attributed to the presence of C3’-4’ OH-groups [23]. Thus, quercetin showed the highest antioxidant activity among the tested flavonoids followed by isoquercitrin. The results were in agreement with previous studies describing the antioxidant potentials of phenolic compounds that resulted in the strong antioxidant activity of the PF [24].

### Inhibitory Activity of the Isolated Compounds (P1-P6) Against Key Enzymes Related to MS

The six compounds showed a powerful inhibitory activity against the enzymes with IC_50_ values ranging from 0.048 to 47.80 µM (Table S7). It has been demonstrated that dietary polyphenols effectively inhibit MS-related enzymes. Their ability to hydrogen bond with proteins has been linked to this function [15]. Among the tested compounds, cinnamic acid (P6) showed the best in vitro inhibitory activity against all the tested enzymes. Cinnamic acid showed a comparable inhibitory activity against *α*-glucosidase (0.75 µM) and ACE (0.048 µM) to the respective reference drug (0.74 and 0.040 µM, respectively). The mechanism by which the phenolic compounds inhibit *α*-glucosidase and *α*-amylase was correlated with their hydroxyl groups [25]. In addition, phenolic compounds exhibited a structure-function relationship in inhibiting ACE activity through the chelation of the active site zinc ion and the development of hydrogen bridges between the active site amino acid residues and the phenols to decrease ACE activity [26]. Regarding the iNOS inhibitory activity, the 6 isolated compounds showed comparable results (4.76–4.99 µM) to the reference drug parthenolide (4.51 µM). These results are justified by previous studies exploring the antidiabetic and antihypertensive activities of phenolic compounds found in edible fruits [24]. This work emphasizes the antidiabetic and antihypertensive activities of the isolated compounds and confirms their potential in the management of MS.

### Molecular Dynamic and System Stability

Molecular dynamic simulations were performed to gain insight into how the observed enzyme inhibitory activity of the isolated compounds may be translated into therapeutic outcomes. To track disrupted motions and prevent artifacts that can appear during the simulation course, the system stability must be validated. For shedding light on lead compounds with inhibitory activities against MS-related enzymes comparable to the reference standard, cinnamic acid was a suitable candidate for *α*-glucosidase and ACE. Therefore, the ligand-protein interactions of cinnamic acid in the active site of the aforementioned enzymes as well as its stability through simulation was predicted. Furthermore, the six isolated compounds exhibited inhibitory activities against iNOS nearly similar to the reference drug. Therefore, the six compounds were docked into the active site of iNOS to clarify their inhibitory potential.

### *In Silico* Evaluation of Cinnamic Acid (P6) Against *α*-glucosidase and *α*-amylase

This study assessed Root-Mean-Square Deviation (RMSD) to measure the systems’ stability during the 20 ns simulations. The recorded average RMSD values for all frames of systems apo-protein, and cinnamic acid-complex system for the *α*-glucosidase were 1.49 Å and 1.37 Å, respectively (Fig. S4A). While those of systems apo-protein, and cinnamic acid-complex system for the ACE were 1.67 Å and 1.33 Å, respectively (Fig. S5A). These results revealed that the cinnamic acid-bound to protein complex system acquired a relatively more stable conformation than the other studied system on both targets. Using the Root-Mean-Square Fluctuation (RMSF) technique, protein residue variations were assessed to determine the impact of inhibitor binding to the relevant targets across 20 ns of simulations. The computed average RMSF values were 5.94Å, and 1.02 Å for apo-protein, and cinnamic acid-complex systems for the *α*-glucosidase, respectively. Moreover, the computed average RMSF values were 4.56Å, and 0.93 Å for apo-protein, and cinnamic acid-complex system for the ACE, respectively. Overall residue fluctuations of individual systems are represented in Fig. S4B and S5B. These values revealed that the cinnamic acid-bound to protein complex system has a lower residue fluctuation than the other systems on both targets. The protein structure compactness, as well as stability during the simulation, were determined by the radius of gyration (Rg). As it was shown in Fig S4C, Rg values of apo-protein and complex with cinnamic acid were 24.26 Å and 24.15 Å for the *α*-glucosidase. While those of apo-protein and complex with cinnamic acid were 24.54 Å and 24.48 Å for the ACE (Fig. S5C). It was found that Rg of ligand-bonded protein exhibited a lower rigid structure than apo-protein.

### Binding Interaction Mechanism Based on Binding Free Energy Calculation

The molecular mechanics/generalized Born surface area (MM-GBSA) program in AMBER18 was used to calculate the binding free energies by extracting snapshots from the trajectories of the systems. All the reported calculated energy components [except solvation free energy (ΔG_solv_)] gave high negative values indicating favorable interactions. The results indicated that the binding affinity of cinnamic acid-bonded to *α*-glucosidase and ACE were − 23.11 kcal/mol and − 20.03 kcal/mol, respectively (Table S8). The interactions between the cinnamic acid and the *α*-glucosidase and ACE protein receptors residues are driven by the more positive electrostatic energy components, as shown by a detailed examination of each energy contribution, leading to the reported binding free energies.

### *In Silico* Evaluation of the Isolated Compounds (P1-P6) Against iNOS

The recorded average RMSD values for all frames of systems apo-protein, rutin-complex systems, isoquercitrin-system, isorhamnetin-3-*O*-*β*-D-glucopyranoside-system, cinnamic acid-system, quercetin-system, and chlorogenic acid-system were 1.50 ± 0.19 Å, 1.64 ± 0.42 Å, 1.54 ± 0.23 Å, 1.68 ± 0.15Å, 1.77 ± 0.19 Å 1.48 ± 0.13 Å, and 2.22 ± 0.57 Å (Fig. S6A). The computed average RMSF values were 7.69 ± 3.06 Å, 5.23 ± 1.84Å, 5.36 ± 1.75Å, 1.18 ± 1.02Å, 1.00 ± 0.55Å, 0.99 ± 0.64Å, and 4.76 ± 0.8Å for apo-protein, cinnamic acid-system, isoquercitrin-system, isorhamnetin-3-*O*-*β*-D-glucopyranoside-system, rutin-complex systems, quercetin-system, and chlorogenic acid-system, respectively (Fig. S6B). These results revealed that the quercetin-bound to protein complex system acquired a relatively more stable conformation than the other studied systems.

### Binding Interaction Mechanism Based on Binding Free Energy Calculation

All the reported calculated energy components (except ΔGsolv) gave high negative values indicating favorable interactions. The results indicated that the binding affinity of cinnamic acid-system, chlorogenic acid-system, quercetin-system, isoquercitrin-system, isorhamnetin-3-*O*-*β*-D-glucopyranoside-system, and rutin-complex system were − 13.47 kcal/mol, -22.98 kcal/mol, -31.13 kcal/mol, -39.43 kcal/mol, -43.80 kcal/mol, and − 68.85 kcal/mol, respectively (Table S9). The interactions between the tested compounds and the iNOS protein receptor residues are driven by the more positive electrostatic energy component, as shown by a detailed examination of each energy contribution, leading to the reported binding free energies (Table S9).

### Identification of the Critical Residues Responsible for Ligands Binding

The total energy when cinnamic acid binds to both *α*-glucosidase and ACE enzyme receptors is represented in Fig. S7. Finally, the total energy involved when rutin, isoquercitrin, isorhamnetin-3-*O*-*β*-D-glucopyranoside, chlorogenic acid, quercetin, and cinnamic acid bind these enzymes was further decomposed into the involvement of individual site residues to get more knowledge about important residues involved in the inhibition of the iNOS receptor (Fig. S8).

## Conclusions

This study highlighted *P. pyrifolia* as a promising fruit to improve MS-related alterations for the first time. Where, HPLC-UV analysis provided a deep insight into the phenolic composition of the fruit extract and allowed the quantification of 15 phenolic compounds, mostly with previously reported antidiabetic and hypertensive potential. Furthermore, the proceeded biologically guided fractionation led to the isolation of *Pyrus* major constituents, which through deep in vitro investigation against different key enzymes for metabolic disorders, revealed potent potential against diabetes, obesity, and hypertension which are the main risk factors associated with the metabolic abnormalities related to MS. Finally, the binding modes of the isolated compounds with *α*-glucosidase and ACE, as well as iNOS were determined suggesting that they could be promising leads against MS. Our future perspective is to support our in vitro and *in silico* studies with in vivo testing for holistic evaluation of *P. pyrifolia* and its isolates as a functional food with beneficial effect against metabolic syndrome.

## Electronic Supplementary Material

Below is the link to the electronic supplementary material.

Supplementary Material 1

## Data Availability

All data generated or analyzed during this study are included in this published article (and its Supplementary Information file).

## Abbreviations

ABTS: 2,2'-azino-di(3-ethylbenzthiazoline-6-sulfonic acid
ACE: angiotensin I converting enzymes
DW: dried weight
FRAP: ferric reducing antioxidant power
GAE: gallic acid equivalent
iNOS: inducible nitric oxide synthase
ME: methanolic extract
NO: nitric oxide
NPF: non-polar fraction
ORAC: oxygen radical absorbance capacity
PF: polar fraction
QE: quercetin equivalent
TE: Trolox equivalent
TFC: total flavonoid content
TPC: total phenolic content
XO: xanthine oxidase
