# Supplementary material for: Modulatory Effect of Pyrus pyrifolia Fruit and its Phenolics on Key Enzymes against Metabolic Syndrome: Bioassay-Guided Approach, HPLC Analysis, and In Silico Study
Source: Plant Foods Hum Nutr. 2023 May 23;78(2):383–9. doi: 10.1007/s11130-023-01069-3 (PMC10363080; doi:10.1007/s11130-023-01069-3)
Supplement: Supplementary file 1 — Supplementary Material 1 [file 11130_2023_1069_MOESM1_ESM.pdf]

Supplementary Material

Modulatory Effect of *Pyrus pyrifolia* Fruit and its Phenolics on Key Enzymes against Metabolic Syndrome: Bioassay-Guided Approach, HPLC Analysis, and *In Silico* Study

Nariman E. Mahdy[1]†, Passent M. Abdel-Baki[1]†*, Ahmed A. El-Rashedy[2], Rana M. Ibrahim[1]

[1]Pharmacognosy Department, Faculty of Pharmacy, Cairo University, Kasr-El-Ainy Street, 11562, Cairo, Egypt

[2]Natural and Microbial Products Department, National Research Center (NRC), Egypt.

***Corresponding author: Passent M. Abdel-Baki, passent.mohamed@pharma.cu.edu.eg

† These authors contributed equally to this work.

Materials and Methods

General procedures

Polyamide, silica gel 60 (70–230 mesh), silica gel RP-18, and Sephadex LH-20 were used. Thin layer chromatography (TLC) was performed on precoated silica gel 60 F_{254} plates (Sigma-Aldrich Chemicals, Germany). Solvent systems employed for TLC development were S_1: methylene chloride-methanol-formic acid (90:10:0.2 v/v/v); S_2: methylene chloride-methanol-formic acid (85:15:0.2 v/v/v); S_3: ethyl acetate-formic acid-glacial acetic acid-water (100:11:11:10 v/v/v/v); S_4: *n*-butanol-acetic acid-water (4:1:2 v/v/v, upper phase). Chromatograms were visualized under UV light (254 and 366 nm) before and after ammonia vapor exposure and spraying with aluminum chloride and after spraying with *p*-anisaldehyde–sulphuric acid, followed by heating at 110 °C. Paper chromatography was performed on Whatmann No. 1 filter paper (Whatmann, Ltd., England) using solvent system S_4, *n*-butanol-acetic acid-water (4:1:2 v/v/v, upper phase) and visualized by spraying with aniline phthalate spray reagent. Solvents used for extraction and fractionation were

all of analytical grade. An electrothermal 9100 (UK) was used for the determination of melting points (uncorrected). A Jenway model 6800 spectrophotometer was utilized for recording UV spectra. A Tecan, microplate reader, ((Infinite F50, Switzerland)) was used to measure the absorbances. A Bruker NMR system was used for ^{1}H-NMR (400 MHz) and ^{13}C-NMR (100 MHz). The NMR spectra were determined in CD_3OH and DMSO-d6. Chemical shifts are given in δ (ppm) relative to the internal standard TMS. Phenolic acids and flavonoids used in HPLC analysis as well as all other reagents for biological investigations were obtained from Sigma Chemical Company (CA, USA).

Determination of total phenolic (TPC) and flavonoid (TFC) contents of *P. pyrifolia* fruits methanolic extract (ME), non-polar (NPF), and polar (PF) fractions

The TPC was determined by using the Folin–Ciocalteu method according to the procedure described previously [1] and was calculated as gallic acid equivalent (GAE). While the TFC was evaluated by using $AlCl_3$ method [2] and was expressed as quercetin equivalent (QE).

Determination of the antioxidant activity

2,2`-Azino-bis(3-ethylbenzothiazoline-6-sulfonic acid) (ABTS), ferric reducing antioxidant power (FRAP), and oxygen radical absorbance capacity (ORAC) assays were performed according to the previously published procedures [3-5]. Ascorbic acid was used as a reference antioxidant drug. Results were expressed as micromolar (μM) Trolox equivalent (TE) per gram of tested sample (i.e., μM TE/g).

Inhibitory activity against key enzymes related to metabolic syndrome (MS)

All assays were performed in 96-well plates. Stock solutions of the extract (100 mg/mL), fractions (100 mg/mL), and isolated compounds (1000 µM) were prepared. Tested samples that exceeded 50% inhibition at these concentrations were serially diluted to determine their half-maximal

inhibitory concentration (IC_{50}). The percentage of enzyme inhibition was calculated according to the equation:

$$\% \text{ Inhibition} = \frac{(A-B)-(C-D)}{(A-B)} \, x \, 100$$

A is the activity of the enzyme without tested samples, B is the control of A with neither tested samples nor enzyme, C, and D denote the activity of the test solutions with and without the tested enzyme, respectively.

α-Glucosidase inhibitory activity

α-Glucosidase inhibition assays were performed according to a previously described method [6]. The tested samples were mixed with glutathione, α-glucosidase solution *(Saccharomyces cerevisiae*, EC 3.2.1.20, Sigma) in phosphate buffer, and the chromogenic substrate *p*-nitrophenyl-D-glucopyranoside (pNPG). Tested samples (50 µL) were incubated with α-glucosidase (100 µL. 0.6 U mL^{-1}) in the presence of phosphate buffer (0.1 M, pH 6.9) for 10 min at 37 °C. Fifty µL of 3nM pNPG in phosphate buffer (pH 6.9) was added, and the mixture was incubated again for 12 min at 37 °C. Enzyme activity was evaluated by measuring ρ-nitrophenol release from the ρNPG substrate. acarbose was used as a reference drug. Absorbance at 405 nm was measured.

α-Amylase inhibitory activity

α-Amylase inhibition assays were performed according to a previously described method [6]. The tested samples were incubated with pancreatic α-amylase (ex-porcine pancreas, EC 3.2.1.1, Sigma) followed by the addition of starch and iodine-potassium iodide solution for color development [6]. Starch [50 µL, 1% phosphate-buffered saline (PBS)] was mixed as substrate with 13 U mL^{-1} pancreatic α-amylase prepared in PBS, and 50 µL of test solution. A blank was performed using PBS. To initiate the reaction, the enzyme was added to the assay solution after being pre-incubated separately at 37 °C in a water bath for 10 min. Dinitrosalicylic acid reagent (1 mL) was added and

heated at 85 °C for 15 min. Then, it was cooled to room temperature and distilled water (1 mL) was added. Acarbose was used as a reference drug. The absorbance was recorded at 540 nm.

Pancreatic lipase inhibitory activity

The pancreatic lipase inhibitory activity was assessed using *p*-nitrophenyl dodecanoate (p-NPD) as substrate and porcine pancreatic lipase (Abnova, Taipei, Taiwan) which was described previously [7]. Orlistat was used as a reference drug. The tested samples were diluted in DMSO were incubated with 10 g L−1 PL (diluted in 0.05 mol L−1 Tris-HCl buffer pH 8.0, containing 0.010 mol L−1 $CaCl_2$ and 0.025 mol L−1 NaCl) for 20 min at 37 °C. p-NPP (0.008 mol/L, diluted in 0.5% TritonX 100) (m v−1) was added to initiate the reaction. The absorbances were measured at 410 nm for 30 min at 37 °C.

Angiotensin conversion enzyme (ACE) inhibitory activity

Assessment of ACE inhibitory activity was performed using an earlier published method [8] using ACE1 solution (EC: 3.4.15.1, Biovision, California, United States) and histidine-l-hippuryl-l-leucine-chloride (HHL) as a chromogenic synthetic substrate. Zofenopril was used as a reference drug. Forty µL of the enzyme solution (2 mU ACE produced in 0.1-M Na borate buffer) was mixed with 20 µL of each tested dilution of each sample and incubated at 37°C for 10 minutes, followed by the addition of 40 µL HHL substrate (0.8 mM/L) and incubation for 1 hour at 37 °C. Sixty µL of 0.5 M sodium hydroxide was added to stop the process. The blank solution for each sample was prepared by substituting the buffer solution for the enzyme solution. Methanol was used to make the control solutions in place of the sample. Fluorescence was measured at excitation (360 nm) and emission wavelengths (500 nm).

Renin inhibitory activity

The renin inhibitory activity was examined according to a previously described method [9] using renin-inhibitor screening assay kit (BPS Bioscince, San Diego, CA, USA) and quinapril as a reference drug. Recombinant renin enzyme (50 µL) was dissolved in 50-mM Tris-HCl buffer (pH 8.0) and 100-mM NaCl (assay buffer) and stored at –80°C for further analysis. Twenty µL of renin substrate (A500 µM in DMSO), 150 µL of assay buffer, and 10 µL of each sample were used to prepare test solutions (10mg/ml in methanol). The blank samples were made using 10 µL of the sample, 20 µL of the substrate, and 160 µL of assay buffer. The positive control samples were created using 10 µL of methanol, 20 µL of substrate, and 150 µL of assay buffer. The positive control and tested samples were combined with 10 µL of the renin solution for the assay in order to catalyze the reaction. After that, the reaction mixture was incubated for 45 minutes at 37 °C. The fluorescence generated at the 340 nm excitation and 490 nm emission wavelengths was measured using a microplate reader.

Xanthine oxidase (XO) inhibitory activity

Xanthine oxidase (XO) inhibitory potential was carried out according to a previous designed procedure [10] using xanthine oxidase (XO, EC 1.17.3.2; Biovision, USA). Allopurinol was applied as a reference drug. Immediately prior to use, a mixture was made up of 50 µL of sample solution, 35 µL of phosphate buffer (70 mM, pH = 7.5), and 30 µL of new enzyme solution (0.01 units/mL in the same buffer). The reaction was started by adding 60 µL of the substrate solution (150 µM xanthine in the same buffer) following a pre-incubation at 25 °C for 15 minutes. After that, the mixture was incubated at 25 °C for 30 min. After adding 25 µL of 1 M HCl to terminate the reaction, the absorbance was measured at 290 nm with a microplate reader as previously

mentioned. The same procedure was used to prepare a blank, except after pipetting HCl, the enzyme solution was added.

Inducible nitric oxide synthase (iNOS) inhibitory activity

iNOS inhibitory activity was determined in mouse macrophage cells (RAW264.7, Shanghai BOGO Industrial Co., Ltd., China) according to a previously designed method [11]. Phenol red-free RPMI medium from Thermo-Fisher was used to develop macrophages. It was supplemented with 10% bovine calf serum, 100 U/mL of penicillin G sodium from Sigma-Aldrich, and 100 g/mL of streptomycin (Sigma-Aldrich). Cells (50,000/well) were seeded onto 96-well plates, which were then incubated for 24 hours. The cells were treated with the tested samples for 30 min. The cells were induced by lipopolysaccharide (LPS) (Sigma-Aldrich) (5 g/mL), and the incubation process lasted for 24 hours. By measuring the amount of nitrite in the cell culture supernatant using Griess reagent, the activity of iNOS (Sigma-Aldrich, St. Louis, MO) was calculated in terms of NO concentration. Cell viability was determined with MTT reduction assay [12]. Absorbances were measured at 540 nm using a microplate reader. The positive control was parthenolide.

High performance liquid chromatography (HPLC) analysis of *P. pyrifolia* fruits polar fraction (PF)

HPLC analysis was performed on Agilent 1260 infinity HPLC Series (Agilent, USA), equipped with Quaternary pump, aKinetex®5μm EVO C18 100 mm x 4.6 mm column (Phenomenex, USA), operated at 30 °C using a ternary linear elution gradient with (A) HPLC grade water 0.2 % H_3PO_4 (v/v), (B) methanol, and (C) acetonitrile with flow rate 0.2 mL/min. A variable wavelength detector (VWD) set at λ 280 nm was used (Agilent Application Note, Publication number 5991-3801EN, 2014). A ternary linear elution gradient with (A) HPLC grade water 0.2 % H_3PO_4 (v/v), (B) methanol, and (C) acetonitrile with flow rate 0.2 mL/min was adopted. The operation

temperature was 30 °C. A variable wavelength detector (VWD) set at λ 280 nm was used (Agilent Application Note, Publication number 5991-3801EN, 2014). Three biological replicates were analyzed. Comparing retention times of the peaks with those of the standard phenolics allowed for qualitative determination, whereas peak area measurement allowed for quantitative determination.

Molecular Docking Analysis

Molecular docking study was performed on the isolated compounds to study the mode of their interaction in the active site of the yeast isomaltase from *Saccharomyces cerevisiae* (Pdb id:3A4A) with 84% similarity to *S. cerevisiae α*-glucosidase, human angiotensin-converting enzyme (ACE) (Pdb id: 2XYD), and nitric oxide synthase (NOS) enzyme (Pdbid: 3E7G) [13-15] using Auto Dock Tools (version 1.5.6). These structures were then prepared for molecular dynamics (MD) studies using UCSF Chimera [16]. Using PROPKA, pH was fixed and optimized to 7.5 [17]. The structures of compounds (P1-P6) were drawn using ChemBioDraw Ultra 12.1 [18]. For *α*-glucosidase, ACE and NOS all prepared systems were subjected to 20 ns MD simulations, as detailed in the simulation section.

Molecular dynamic (MD) simulations

The application of molecular dynamic (MD) simulations in the study of biological systems allows for the exploration of physical motions of atoms and molecules that are not easily accessible by other techniques [19]. The understanding gained from running this simulation provides a detailed look into the dynamical evolution of biological systems, such as conformational changes and molecule interaction [19]. The MD simulations of all systems were carried out using the GPU version of the PMEMD engine included in the AMBER 18 package [20].

The partial atomic charge of each compound was calculated with ANTECHAMBER's General Amber Force Field (GAFF) technique [21]. The Leap module of the AMBER 18 package

implicitly solvated each system within an orthorhombic box of TIP3P water molecules within 10 Å of any box edge. The Leap module was used to neutralize each system by incorporating Na^+ and Cl^- counter ions. A 2000-step initial minimization of each system was carried out in the presence of a 500 kcal/mol applied restraint potential, followed by a 1000-step full minimization using the conjugate gradient algorithm without restraints.

During the MD simulation, each system was gradually heated from 0K to 300K over 500ps, ensuring that all systems had fixed number of atoms and fixed volume. The system's solutes were subjected to a 10kcal/mol potential harmonic constraint and a 1ps collision frequency. Following that, each system was heated and equilibrated for 500ps at a constant temperature of 300K. To simulate an isobaric-isothermal (NPT) ensemble, the number of atoms and pressure kept constant within each system for each production simulation, with a stable system's pressure at 1 bar using the Berendsen barostat [22].

For 20 ns, each system was MD simulated. The SHAKE method was used to constrain the hydrogen bond atoms in each simulation. Each simulation used a 2fs step size and integrated an SPFP precision model. An isobaric-isothermal ensemble (NPT) with randomized seeding, constant pressure of 1 bar, a pressure-coupling constant of 2ps, a temperature of 300K, and a Langevin thermostat with a collision frequency of 1ps was used in the simulations.

Post-MD Analysis

After saving the trajectories obtained by MD simulations every 1 ps, the trajectories were analyzed using the AMBER18 suite's CPPTRAJ [23] module. The Origin [24] data analysis program and Chimera [16] were used to create all graphs and visualizations.

The Poisson-Boltzmann or generalized Born and surface area continuum solvation (MM/PBSA and MM/GBSA) approach has been found to be useful in the estimation of ligand-binding affinities

[25-27]. The Protein-Ligand complex molecular simulations used by MM/GBSA and MM/PBSA compute rigorous statistical-mechanical binding free energy within a defined force field. Binding free energy averaged over 200 snapshots extracted from the entire 20 ns trajectory. The estimation of the change in binding free energy (ΔG) for each molecular species (complex, ligand, and receptor) can be represented as follows [28]:

$$\Delta G_{bind} = G_{complex} - G_{receptor} - G_{ligand} \quad (1)$$

$$\Delta G_{bind} = E_{gas} + G_{sol} - TS \quad (2)$$

$$E_{gas} = E_{int} + E_{vdw} + E_{ele} \quad (3)$$

$$G_{sol} = G_{GB} + G_{SA} \quad (4)$$

$$G_{SA} = \gamma SASA \quad (5)$$

The terms Egas, Eint, Eele, and Evdw symbolize the gas-phase energy, internal energy, Coulomb energy, and van der Waals energy. The Egas was directly assessed from the FF14SB force field terms. Solvation free energy (Gsol) was evaluated from the energy involvement from the polar states (GGB) and non-polar states (G). The non-polar solvation free energy (GSA) was determined from the Solvent Accessible Surface Area (SASA) [29, 30] using a water probe radius of 1.4 Å. In contrast, solving the GB equation assessed the polar solvation (GGB) contribution. Items S and T symbolize the total entropy of the solute and temperature, respectively.

Statistical analysis

All determinations were performed in triplicate and were represented as mean ± standard deviation. Microsoft Excel® was used to analyze all IC_{50} data, and GraphPad Prism 8® was used to calculate the IC_{50} values by converting the concentrations to their logarithmic value and then choosing non-

linear inhibitor regression equation (log inhibitor) *vs* normalized response-variable slope equation. One-way ANOVA was used for the statistical analysis, followed by Tukey's test and p values <0.05 were considered significant.

Identification of the critical residues responsible for ligands binding

The total energy involved when rutin, isoquercitrin, isorhamnetin-3-*O*-*β*-D-glucopyranoside, cinnamic acid, quercetin, and chlorogenic acid binds these enzymes was further decomposed into the involvement of individual site residues in order to get more knowledge about important residues involved in the inhibition of the NOS receptor. From Fig. S8, the major favorable contribution of rutin to NOS receptor is mainly observed from residues Met 38 (-1.714 kcal/mol), Trp 121 (-0.39 kcal/mol), Cys 118 (-0.637 k cal/mol), Ile 119 (-0.55 kcal/mol), Ala 180 (-0.34 k.cal/mol), Gln 181 (-2.23 kcal/mol), Ile 183 (-4.59 kcal/mol), Arg 184 (-0.935 kcal/mol), Trp 264 (-0.77 kcal/mol), Tyr 265 (-1.02 kcal/mol), Pro 268 (-1.88 kcal/mol), Ala 269 (-2.26 kcal/mol), Val 270 (-2.23kcal/mol), Asn 272 (-1.22 k.cal /mol), Phe287 (-0.633 kcal/mol), Tyr 291 (-1.048 kcal/mol), Met 292 (-0.867 kcal/mol), Glu 295 (-3.37 kcal/mol), Ile 296 (-1.35 kcal/mol), Arg 299 (-0.624 kcal/mol), and Asp 300 (-4.269 kcal/mol). In addition, the major favorable contribution of isoquercitrin to NOS receptor is observed from residues Met 38 (-0.70 kcal/mol), Arg 117 (-1.60 kcal/mol), Cys 118 (-0.984 kcal/mol), Ile 119 (-1.17 kcal/mol), Gln 181 (-0.491 kcal/mol), Pro 268 (-1.13 kcal/mol), Ala 269 (-0.629 kcal/mol), Ser 22 (-0.903 kcal/mol), Val 270 (-0.98 kcal/mol), Tyr 291 (-1.14 kcal/mol), Met 292 (-0.90 kcal/mol), Glu 295 (-2.403 kcal/mol), Asp 300 (-0.419 kcal/mol), Trp 381 (-2.74 kcal/mol), and Leu 382 (-0.665 kcal/mol). On the other hand, the major favorable contribution of isorhamnetin-3-*O*-*β*-D-glucopyranoside to NOS receptor is obvious from residues Met 38 (-1.95 kcal/mol), Gln 181 (-2.464 kcal/mol), Ile 183 (-0.44 kcal/mol), Asp 198 (-1.12 kcal/mol), Trp 264 (-0.47 kcal/mol), Tyr 265 (-1.46 kcal/mol), Pro 268 (-1.27 kcal/mol), Ala

269 (-0.756 kcal/mol), Val 270 (-0.593 kcal/mol), Tyr 291 (-1.607 kcal/mol), Glu 295 (-0.735 kcal/mol), Ile 296 (-0.316 kcal/mol), and Asp 300 (-5.57 kcal/mol). Finally, the major favorable contribution of cinnamic acid to NOS receptor is predominantly observed from residues Gln181 (-0.53 kcal/mol), Arg 184 (-0.52 kcal/mol), Gln 189 (- 0.196 kcal/mol), Ile 195 (-0.58 kcal/mol), Asp 198 (-0.25 kcal/mol), Pro 199 (-0.39 kcal/mol), Trp 264 (-0.213 kcal/mol), Tyr 265 (-0.463 kcal/mol), Pro 268 (-0.283 kcal/mol), Val 270 (-0.329 kcal/mol), Tyr 291 (-0.251 kcal/mol), Asp300 (-0.675 kcal/mol), and Arg 306 (-0.42 kcal/mol). The major favorable contribution of chlorogenic acid to NOS receptor is obvious from residues Ile 37 (-0.49 kcal/mol), Met 38 (-2.459 kcal/mol), Arg 117 (- 3.65 kcal/mol), Glu295 (-0.43kcal/mol), Arg 299 (-2.64 kcal/mol), Asp 300 (-2.11 kcal/mol), Gln 305 (-0.997 kcal/mol), Arg 306 (-0.44 kcal/mol), and Trp 381 (-1.22 kcal/mol). Finally, the major favorable contribution of quercetin to NOS receptor is obvious from residues Met 38 (-0.627 kcal/mol), Glu 295 (-0.82 kcal/mol), Ile296 (-0.32 kcal/mol), Arg 299 (-1.735 kcal/mol), Asp 300 (-1.868 kcal/mol), Ile 380 (-1.389 kcal/mol), Trp 381 (-2.681 kcal/mol), Val 383 (-0.707 kcal/mol), Pro384 (-0.245 kcal/mol), and Pro385 (-1.118 kcal/mol).

The total energy when cinnamic acid binds ACE receptor enzyme was mainly detected from residues Asp 252 (-0.299 kcal/mol), Gln 256 (-0.34 kcal/mol), Ser 257 (-0.864 kcal/mol), Asp 351 (-0.691 kcal/mol), Thr 355 (-1.379 kcal/mol), Hid 358 (-0.937 kcal/mol), Glu 428 (-2.77kcal/mol), Lys 429 (-0.577 kcal/mol), Phe 342 (-1.743 kcal/mol), Tyr 498 (-0.354 kcal/mol), Phe 502 (-1.019 kcal/mol), and Gln 505(-0.21 kcal/mol).

On the other hand, The total energy when cinnamic acid binds glucosidase receptor enzyme was primarily perceived from residues Asp 66 (-3.515 kcal/mol), Tyr 69 (-1.95 kcal/mol), Asp 70 (-0.397 kcal/mol), Tyr 155 (-0.451 kcal/mol), Phe 156 (-1.00 kcal/mol), Phe 175 (-1.231 kcal/mol),

Phe 212 (-2.73kcal/mol), Val 213 (-0.659 kcal/mol), Hie 348 (-0.452 kcal/mol), Asp 349 (-1.629 kcal/mol), Asp 406 (-0.309 kcal/mol), and Glu 408 (-0.178 kcal/mol).

Supplementary Tables and Figures

Table S1. TPC, TFC and antioxidant activity using ABTS, FRAP and ORAC methods of *P. pyrifolia* fruits methanolic extract (ME), non-polar (NPF) and polar (PF) fractions.

Sample	TPC	TFC	ABTS		FRAP		ORAC	
	µg GAE/mg DW*	µg QE/mg DW*	µM TE/g*	AA (%)	µM TE/g*	AA (%)	µM TE/g*	AA (%)
ME	12.18±0.42	9.11±0.64	230.83±0.60	80.21	105.68 ±0.19	68.08	255.23±0.89	65.41
NPF	7.52±0.18	3.75±0.98	160.69±0.91	55.84	82.02±0.92	52.84	189.28±1.08	48.51
PF	20.28±0.17	16.39±0.52	270.97±0.35	94.16	139.11±0.15	89.62	301.84±2.01	77.35
Ascorbic acid	-	-	287.78±1.01	100	155.22±0.03	100	390.21±0.99	100

*Data are expressed as mean ± standard deviation of three replicates; µM: micromolar; AA(%): antioxidant activity percentage; DW: dry weight; GAE: gallic acid equivalent; ME: methanolic extract; NPF: non-polar fraction; PF: polar fraction; QE: quercetin equivalent; TE: Trolox equivalent; TFC: total flavonoid content; TPC: total phenolic content.

Table S2. Inhibitory activity of the methanolic extract (ME), non-polar (NPF) and polar (PF) fractions against key enzymes related to MS

Enzyme	Inhibition (IC_{50}) µg/mL			
	**ME**	**NPF**	**PF**	**Standard**
α-Glucosidase	9.51±0.21	15.54±0.18	2.24±0.41	^{A} 0.476±0.02
α-Amylase	17.21±0.71	20.12±0.38	4.26 ±0.28	^{A} 0.675±0.02
Lipase	28.9 ±0.67	31.89 ±0.03	15.78 ±0.50	^{B} 0.172±0.01
ACE1	12.90 ±0.89	16.78 ±1.29	9.90 ±0.12	^{C} 0.017 ±0.001
Renin	31.58±1.3	71.56±1.3	17.56 ±0.79	^{D}0.02±0.01
iNOS	18.75±1.94	21.67 ±0.99	12.12±1.04	^{E}1.12±0.91
XO	32.57±0.61	59.67 ±0.14	20.82 ±0.97	^{F}1.07±0.05

Values are expressed as mean ± SD; ^{A}: acarbose; ^{B}: orlistat; ^{C}: zofenopril; ^{D}: quinapril; ^{E}: parthenolide; ^{F}: allopurinol.

Table S3. Physicochemical, chromatographic and UV spectral data of compounds P1-P6

		P1	P2	P3	P4	P5	P6
**Physical properties**	**Condition**	Crystals (190 mg)	Crystals (103 mg)	Crystals (120 mg)	Crystals (105 mg)	Crystals (210 mg)	Crystals (180 mg)
	**Color**	Yellow	Yellow	Yellow	White	Yellow	White
	**Melting range**	241-243 °C	224-226 °C	267-268 °C	206-208°C	310-312°C	132-135 °C
	**Solubility**	Methanol	Methanol	Methanol	Methanol	Methanol	Methanol
**Color reactions**	**UV (λmax 254)**	Deep purple	Deep purple	Deep purple	Blue	Dark yellow	Blue fluorescence
	**UV/NH_3**	Yellow	Yellow	Yellow	Blue fluorescence		Blue fluorescence
	**$AlCl_3$**	Yellow	Yellow	Yellow	Yellow	Yellow	Yellow
**R_f values**	**S_1**	0.18[a], 0.40[c]	0.31[a], 0.70[b]	0.40[a], 0.66[b]	0.55[b]	0.76[a]	0.73[a]
**UV spectral data, λ_{max} (nm)**	**MeOH**	259,301 (sh), 359	258, 274 (sh), 365	255, 349	290, 327	256, 301(sh), 373	270
	**NaOMe**	269,329 (sh), 411	280, 330, 411 (sh)	272. 327 (sh), 411	No change	248(sh), 331, 406	No change
	**$AlCl_3$**	271, 307 (sh), 428	280, 304 (sh), 436	269, 403		269, 456	No change
	**$AlCl_3$/HCl**	269, 299 (sh), 367, 401	277, 366(sh), 409	269, 363(sh), 399		267, 303(sh), 352(sh), 429	No change
	**NaOAc**	265, 301 (sh), 383	279, 329 (sh), 399	268, 319 (sh), 390		268, 329 (sh), 390	No change
	**NaOAc/H_3BO_3**	263, 309 (sh), 379	268, 292 (sh), 391	256, 346		259, 386	No change

P1: rutin; P2: isoquercitrin; P3: isorhamnetin-3-O-*β*-glucopyranoside; P4: chlorogenic acid; P5: quercetin; P6: cinnamic acid; [a]: S_1 [methylene chloride-methanol-formic acid (90:10:0.2 *v/v/v*)]; [b]: S_2 [methylene chloride-methanol-formic acid (85:15:0.2 *v/v/v*)]; [c]: S_3 [ethyl acetate-formic acid-glacial acetic acid-water (100:11:11:10 *v/v/v/v*)].

Table S4. NMR spectral data of compounds P1-P6

	P1[a]		P2 [a]		P3 [a]		P4 [a]		P5 [b]		P6 [b]	
**Position**	^{1}H	^{13}C	^{1}H	^{13}C	^{1}H	^{13}C	^{1}H	^{13}C	^{1}H	^{13}C	^{1}H	^{13}C
**1**		134.36						73.50				
**2**	7.5457 (1H, *m*)	127.86		157.10		156.70		36.22		147.50		156.89
**3**	7.3701 (1H, *m*)	128.64		133.74		133.79	5.0112 (1H, *dd*, *J*=10, 6 Hz)	68.11		136.52		133.33
**4**	7.3701 (1H, *m*)	130.07		177.82		177.90	3.4212 (1H, *br.s*)	70.98		176.51		177.91
**5**	7.3701 (1H, *m*)	128.64		161.66		161.90	3.8721 (1H, *br.s*)	70.33		161 .01		161.77
**6**	7.5457 (1H, *m*)	127.86	6.2088 (1H, *d*, *J*= 1.8 Hz)	99.15	6.2143 (1H, *d*, *J*= 2 Hz)	99.19		37.53	6.1812 (1H, *br.s*)	99.52	6.2841 (1H, *d*, *J*= 1.4 Hz)	99.25
**7**	7.6820 (1H, *d*, *J* = 16 Hz)	145.07		164.53		164.70		175.02		166.01		164.89
**8**	6.4759 (1H, *d*, *J* = 16 Hz)	117.94	6.4025 (1H, *d*, *J*= 1.8 Hz)	94.07	6.4169 (1H, *d*, *J*= 2 Hz)	94.01			6.412 (1H, *d*, *J*=1.5 Hz)	94.52	6.5011 (1H, *d*, *J*= 1.89 Hz)	94.20
**9**				156.88		156.80				156.73		156.72
**10**				104.42		104.50				104.01		104.37
**1'**				121.64		122.10		124.66		123.0		121.56
**2'**			7.5437 (1H, *d*, *J*=1.8 Hz)	116.73	7.6899 (1H, *d*, *J*= 2 Hz)	115.69	7.0221 (1H, *d*, *J*=1.8 Hz)	114.12	7.7412 (1H, *br.s*)	116.5	8.1089 (1H, *d*, *J*=1.43 Hz)	115.78
**3'**				145.99		145.31		145.01		145.7		149.85
**4'**				148.85		149.07		148.88		148.1		147.29
**5'**			6.8683 (1H, *d*, *J*= 8.5 Hz)	115.69	6.8498 (1H, *d*, *J*= 8.4 Hz),	116.72	6.7523 (1H, *d*, *J*=8.4 Hz)	115.66	6.8921 (1H, d, *J*= 8.1 Hz)	116.0	6.9900 (1H, *d*, *J*= 8.46 Hz)	113.90

**6'**	7.5604 (1H, *dd*, *J*= 1.8, 8.5 Hz)	122.06	7.5995 (1H, *dd*, *J*=2, 8.4 Hz),	121.71	6.9521 (1H, *dd*, *J*=8.1, 2 Hz)	121.23	7.6423 (1H, *d*, *J*= 6 Hz)	121.0	7.5611 (1H, *dd*, *J*= 1.57, 8.44 Hz)	122.47
**7'**					7.3522 (1H, *d*, *J*=15.9 Hz)	145.92				
**8'**					6.0721 (1H, d, *J*=16.2 Hz)	114.82				
**9`**						165.65				
**2ax, eq**					2.0852 (2H, *m*)					
**6ax**					1.7322 (1H, *d*, *J*=12.9 Hz)					
**6eq**					1.9022 (1H, dd, *J*=13.8. 10.8 Hz)					
**5-OH**	12.5968								12.6624	
**3'-OCH$_3$**									3.8981 (3H, *s*)	56.15
**1''**	5.3627 (*d*, *J*= 7.08 Hz)	101.63	5.4699 (1H, *d*, *J*=7.8 Hz)	101.38					5.6412 (1H, *d*, *J*= 7.13 Hz)	101.21
**2''**	3.7300-3.0653 (10 H) *	74.52	3.1-3.7 (6H t) *	74.62					3.6715-3.1681 (6H) *	74.82
**3''**	*	76.33		77.18						77.88
**4''**	*	70.45		70.70						70.26
**5''**	*	76.88		78.08						76.86
**6''**	*	68.69		61.56						61.06

**1'''**		4.3990 (*brs*)	101.19
**2'''**		*	71.01
**3'''**		*	70.82
**4'''**		*	72.30
**5'''**		*	67.46
**6'''**		1.0073 (3H, *d*, *J*= 6.12 Hz)	18.17
COOH	169.11		

*Signals are overlapped; [a;] DMSO-d_6; [b]: CD_3OD; ^{13}C (ppm) (100 MHz); ^{1}H δ (multiplicity, *J*/Hz) (400 MHz,); P1: rutin; P2: isoquercitrin; P3: isorhamnetin-3-O-*β*-glucopyranoside; P4: chlorogenic acid; P5: quercetin; P6: cinnamic acid

Table S5. Quantification of some phenolic compounds identified in PF using HPLC

Identified PA	RT (min)	*Conc. (mg /100g ± SD)	Identified F	RT (min)	*Conc. (mg /100g ± SD)
p-Hydroxybenzoic acid	4.761	33.08±0.89	Rutin	10.763	187.90±0.78
Vanillic acid	6.864	11.23±0.74	Isoquercitrin	12.604	353.90±0.33
Caffeic acid	7.424	176.31±0.86	Isorhamnetin-3-*O*-glucoside	12.897	897.75±0.81
Chlorogenic acid	8.022	1888.39±0.58	Quercetin	13.679	2053.94±0.99
p-Coumaric acid	9.196	2.98±0.45	Apigenin	16.902	90.00±0.27
Ferulic acid	9.445	162.94±0.97	Kaempferol	19.398	385.96±0.89
o-Coumaric acid	10.457	38.78±0.35			
Cinnamic acid	13.372	998.79±0.32			
Rosmarinic acid	14.198	40.78±0.28			

*Average concentration of three HPLC determinations, F: flavonoids; PA: phenolic acids; RT; retention time in minutes, SD: standard deviation.

Table S6. Antioxidant activity of the isolated compounds (P1-P6) using ABTS, FRAP and ORAC

Sample	ABTS		FRAP		ORAC	
	**µM TE/g***	**AA (%)**	**µM TE/g***	**AA (%)**	**µM TE/g***	**AA (%)**
**P1**	180.29±1.08	62.64	99.28 ±0.98	63.96	266.30±0.75	68.25
**P2**	240.75±0.28	83.66	137.27±0.53	88.44	346.28±0.29	88.74
**P3**	229.71±0.38	79.82	125.08±0.72	80.58	320.78±0.55	82.21
**P4**	238.22±0.67	82.78	133.63±0.79	86.09	346.16±0.25	89.02
**P5**	244.01±0.29	84.79	139.03±0.65	89.57	358.95±0.78	91.99
**P6**	250.28±0.21	86.97	141.26±0.91	91.01	362.55±1.20	92.91
**Ascorbic acid**	287.78±1.01	100	155.22±0.03	100	390.21±0.99	100

*Data are expressed as mean ± standard deviation of three replicates; AA (%): antioxidant activity percentage; P1: rutin, P2: isoquercitrin, P3: isorhamnetin-3-*O*-*β*-D-glucopyranoside P4: chlorogenic acid; P5: quercetin; P6: cinnamic acid; PF: polar fraction; TE: Trolox equivalent; µM: micromoles.

Table S7. Inhibitory activity of the isolated compounds (P1-P6) against key enzymes related to MS

Enzyme	Inhibition (IC_{50}) µM						
	**P1**	**P2**	**P3**	**P4**	**P5**	**P6**	**Standard**
***α*-Glucosidase**	1.46 ±0.71	1.12±0.38	1.37 ±0.86	1.62 ±0.79	0.78 ±0.25	0.75 ±0.37	^{A}0.74
***α*-Amylase**	3.543±0.21	1.544±0.18	2.310±0.87	3.78±0.19	1.271±0.83	1.017±0.63	^{A}1.05
**Lipase**	0.98 ±0.67	0.57 ±0.03	0.78 ±0.75	1.31±0.29	0.42 ±0.56	0.35 ±0.52	^{B}0.35
**ACE1**	0.42 ±0.78	0.218 ±1.29	0.31 ±0.86	0.57±0.35	0.07 ±0.01	0.048±0.72	^{C}0.040
**Renin**	40.79±1.3	24.79±1.3	34.79±0.79	47.80±0.25	21.92±0.25	20.71±0.8	^{D}0.045
**iNOS**	4.99±1.94	4.80 ±0.99	4.89 ±0.78	4.79±0.89	4.81±0.19	4.76 ±0.25	^{E}4.51
**XO**	4.28±0.61	2.66 ±0.14	3.56 ±0.83	4.79 ±0.09	4.47±0.27	2.12 ±0.35	^{F}7.86

*Data are expressed as mean ± standard deviation of three replicates; ^{A}: acarbose; ^{B}: orlistat; ^{C}: zofenopril; ^{D}: quinapril; ^{E}: parthenolide; ^{F}: allopurinol; ACE, angiotensin I converting enzymes; iNOS: inducible nitric oxide synthase; xanthine oxidase; P1: rutin; P2: isoquercitrin; P3: isorhamnetin-3-O-*β*-D-glucoside; P4: chlorogenic acid; P5: quercetin; P6: cinnamic acid.

Table S8. The calculated energy binding for cinnamic acid against the *α*-glucosidase and ACE protein receptors

Complex	Energy Components (kcal/mol)				
	**ΔE_{vdW}**	**ΔE_{elec}**	**ΔG_{gas}**	**ΔG_{solv}**	**ΔG_{bind}**
**Cinnamic acid-*α*-glucosidase**	-18.49±0.14	-43.59±0.63	-45.76±0.58	43.64±0.62	-23.11±0.16
**Cinnamic acid-ACE**	-23.06 ± 0.10	-27.74 ± 0.70	-29.80± 0.75	27.53± 0.70	-20.03± 0.20

ΔEvdW = van der Waals energy; ΔEele = electrostatic energy; ΔGsolv = solvation free energy; ΔGbind = calculated total binding free energy

Table S9. The calculated energy binding for the rutin, isoquercitrin, isorhamnetin-3-*O*-*β*-D-glucopyranoside, chlorogenic acid, quercetin and cinnamic acid compounds against the NOS protein receptor

	Energy Components (kcal/mol)				
**Complex**	**ΔE$_{vdW}$**	**ΔE$_{elec}$**	**ΔG$_{gas}$**	**ΔG$_{solv}$**	**ΔG$_{bind}$**
Rutin	-57.46± 0.21	-83.03±0.37	-140.49±0.33	71.64± 0.19	-68.85±0.24
Isoquercitrin	-37.69 ±0.28	-66.34 ± 0.95	-14.03 ± 0.75	64.60±0.45	-39.43±0.35
Isorhamnetin-3-*O*-*β*-D-glucopyranoside	-39.94±0.22	-60.04±0.82	-99.98±0.80	56.18±0.54	-43.80±0.33
Chlorogenic acid	-25.66±0.27	-161±0.97	-187.48±0.90	164.50±0.97	-22.98±0.23
Quercetin	-23.79±0.22	-47.78±0.57	-71.58±0.50	40.45±0.24	-31.13±0.36
Cinnamic acid	-13.26 ± 0.16	-17.74 ± 0.78	-31.01 ± 0.87	17.53±0.60	-13.47±0.29

ΔEvdW = van der Waals energy; ΔEele = electrostatic energy; ΔGsolv = solvation free energy; ΔGbind = calculated total binding free energy.

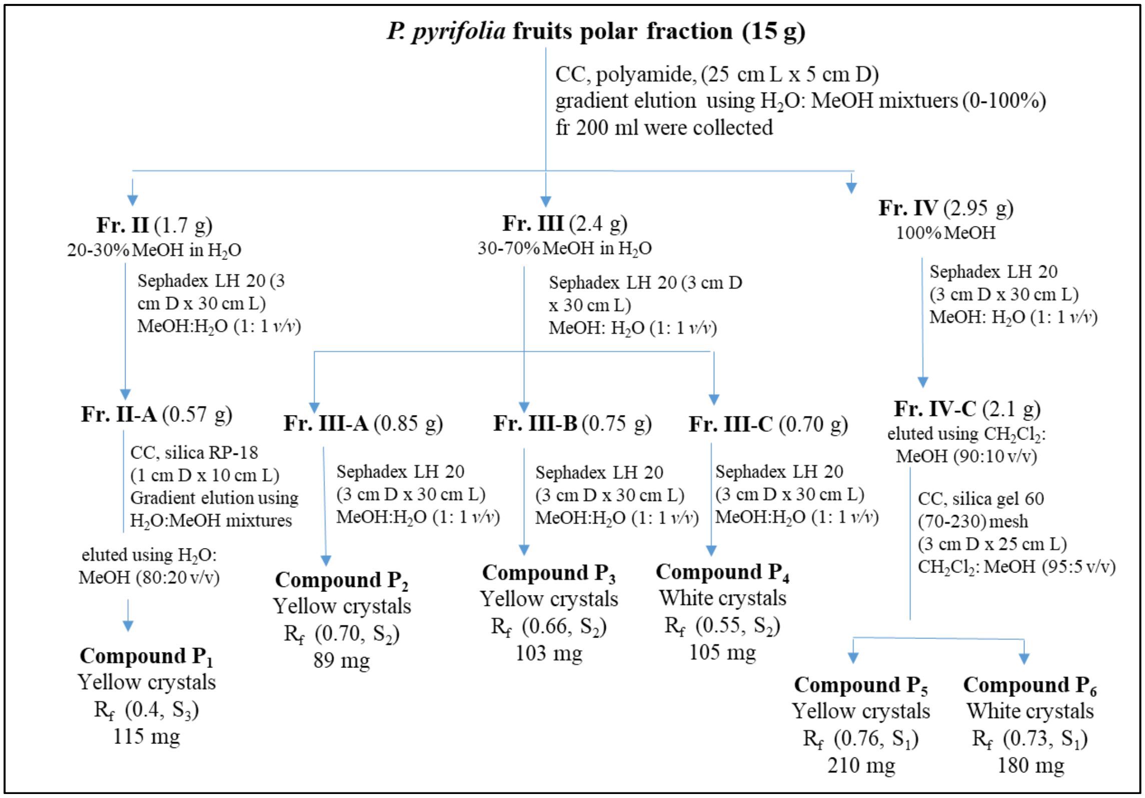

Fig. S1. Scheme for chromatographic fractionation of *P. pyrifolia* fruits polar fraction

Fr.: fraction; MeOH: methanol; S_1: methylene chloride-methanol-formic acid (90:10:0.2 v/v/v); S_2: methylene chloride-methanol-formic acid (85:15:0.2 v/v/v); S_3: ethyl acetate-formic acid-glacial acetic acid-water (100:11:11:10 v/v/v/v).

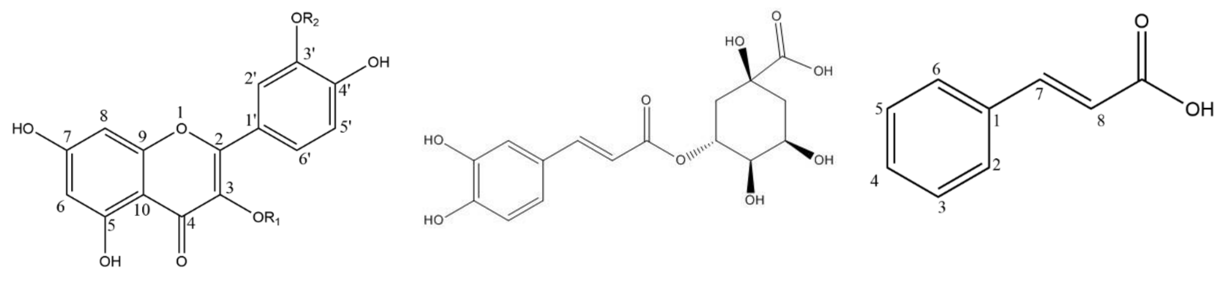

P1: R_1=Glc-Rha, R_2=H
P2 R_1=Glc, R_2=H
P3: R_1=Glc, R_2=CH_3
P5: R_1=H, R_2=H

P4

P6

Fig. S2. Structure of the isolated compounds**, P1**: Rutin; **P2**: Isoquercitrin; **P3**: Isorhamnetin-3-*O*-*β*-D-glucopyranoside; **P4**: Chlorogenic acid; **P5**: Quercetin; **P6**: Cinnamic acid

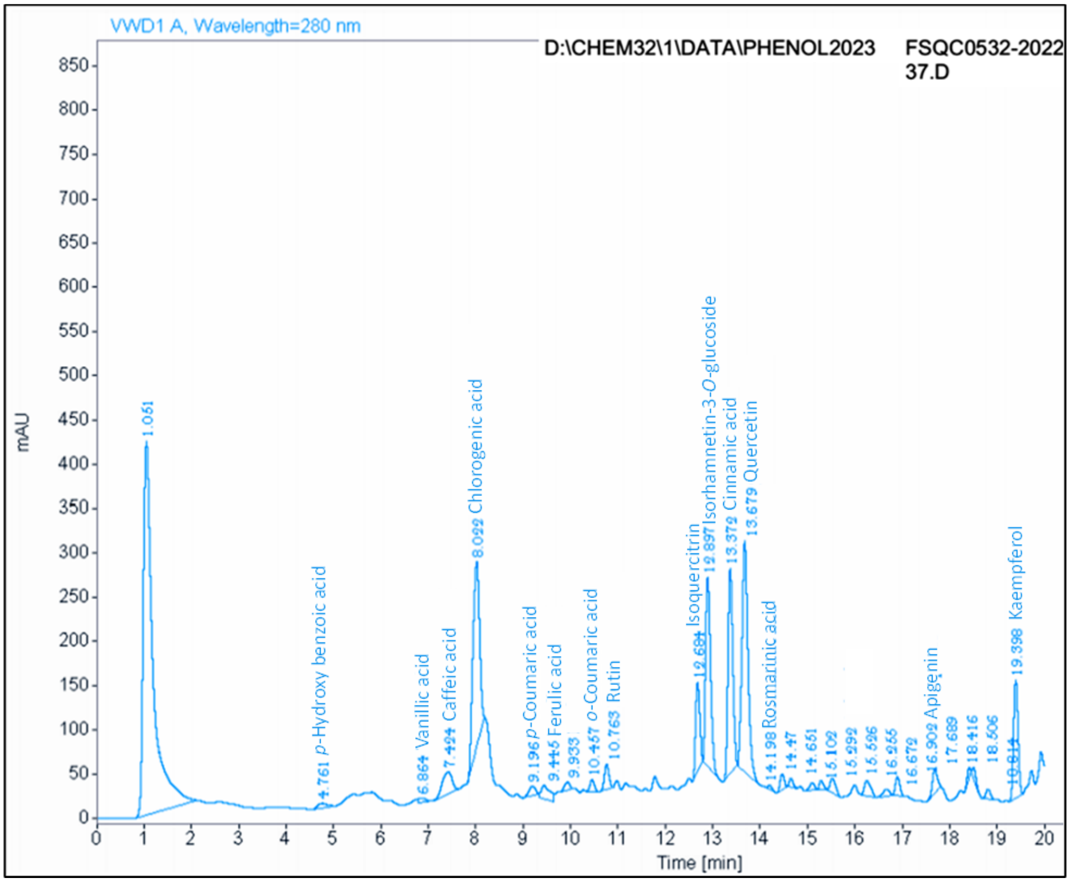

Fig. S3. HPLC chromatogram showing identified phenolic compounds in the *P. pyrifolia* fruits polar fraction (PF) measured at 280 nm.

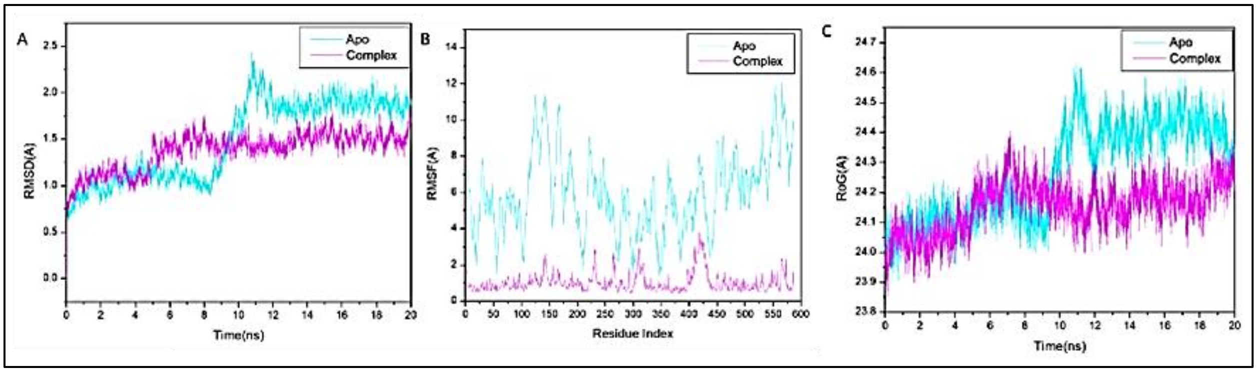

Fig. S4. [A] RMSD of Cα atoms of the protein backbone atoms, [B] RMSF of each residue of the protein backbone Cα atoms, and [C] RoG of Cα atoms of protein residues of the backbone atoms relative (cyn) to the starting minimized over 20 ns for the *α*-glucosidase protein with ligand cinnamic acid (Mag).

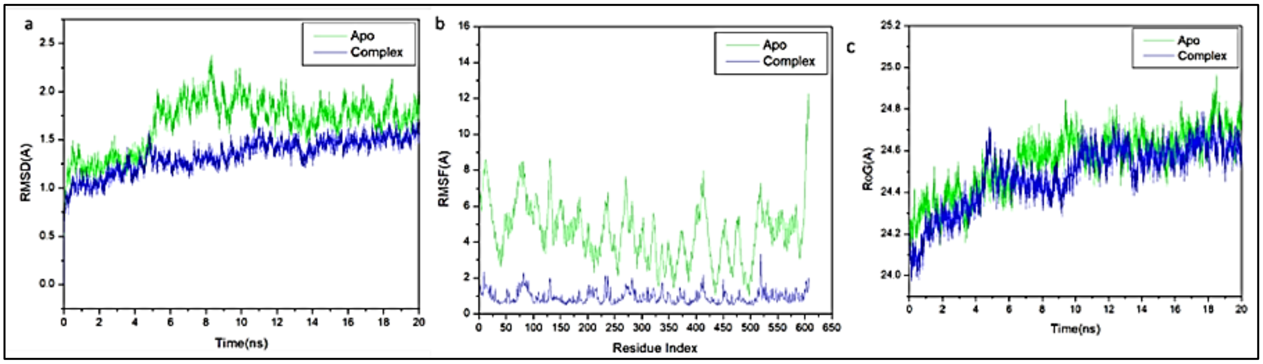

Fig. S5. [A] RMSD of Cα atoms of the protein backbone atoms, [B] RMSF of each residue of the protein backbone Cα atoms, and [C] RoG of Cα atoms of protein residues of the backbone atoms relative (green) to the starting minimized over 20 ns for the ACE protein with ligand cinnamic acid (blue).

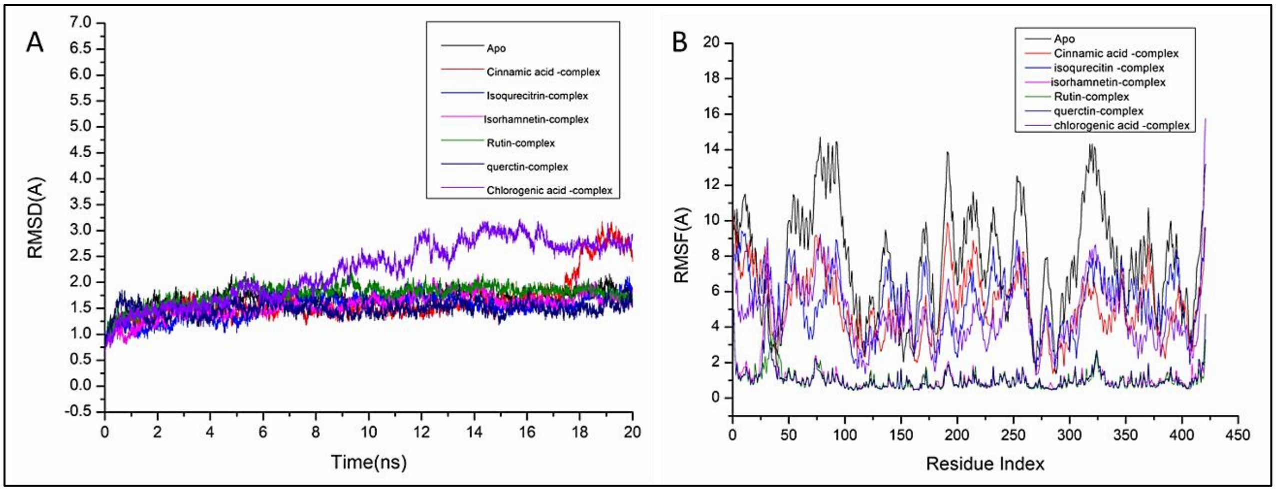

Fig. S6. [A] RMSD of Cα atoms of the protein backbone atoms and [B] RMSF of each residue of the protein backbone Cα atoms of protein residues of the backbone atoms relative (black) to the starting minimized over 20 ns for the NOS protein with ligand rutin (cyn), isoquercitrin (green), isorhamnetin-3-*O*-*β*-D-glucopyranoside (blue), cinnamic acid (red), quercetin-system (navy), and chlorogenic acid-system (violet) .

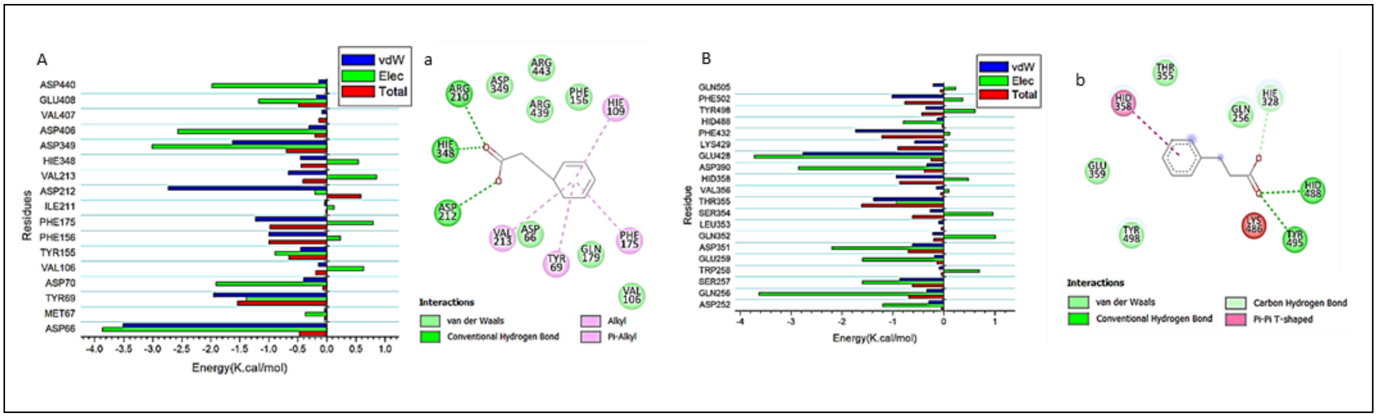

Fig. S7. Per-residue decomposition plots showing the energy contributions of cinnamic acid to the binding and stabilization at the catalytic active site of the [A] α-glucosidase receptor and [B] ACE receptor

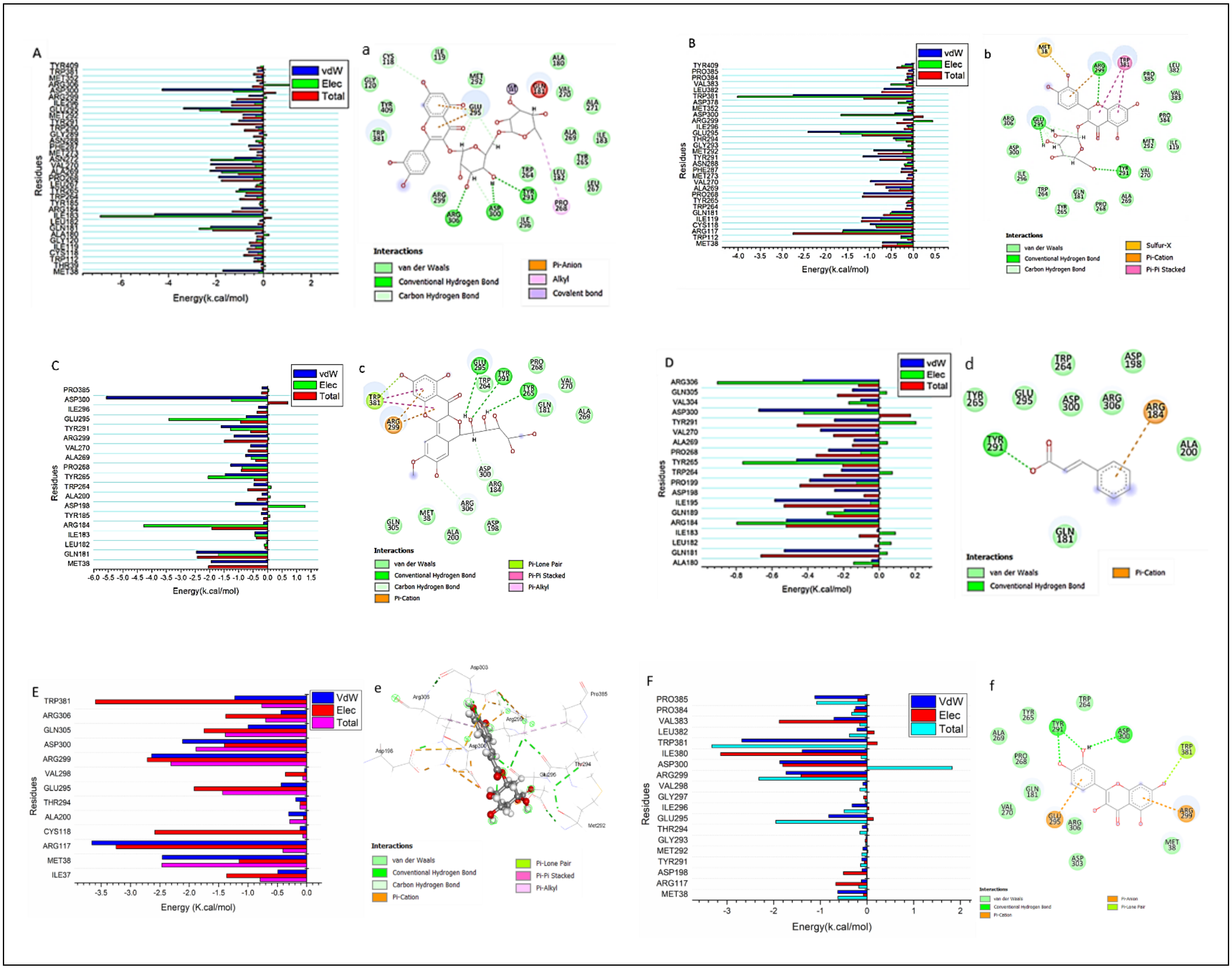

Fig. S8. Per-residue decomposition plots showing the energy contributions to the binding and stabilization at the catalytic active site of the NOS receptor [A] rutin, [B] isoquercitrin, [C] isorhamnetin-3-O-β-D-glucopyranoside, and [D] cinnamic acid [E] chlorogenic acid, and [F] quercetin

References

1. Shweta S, Ritesh T, Khadabadi S, Deokate U (2010) *In vitro antioxidant* activity and total phenolic, flavonoid contents of the crude extracts of *Pterospermum acerifolium* Willd leaves (Sterculiaceae). J Chem Pharm Res 2(3):417-423

2. Kiranmai M, Kumar CM, Mohammed I (2011) Comparison of total flavanoid content of *Azadirachta indica* root bark extracts prepared by different methods of extraction. Res J Pharm, Biol Chem Sci 2(3):254-261

3. Faitanin RD, Gomes JV, Rodrigues PM, de Menezes LFT, Neto ÁC, Gonçalves RC, Kitagawa RR, Silveira D, Jamal CM (2018) Chemical study and evaluation of antioxidant activity and *α*-glucosidase inhibition of *Myrciaria strigipes* O. Berg (Myrtaceae). J Appl Pharm Sci 8(03):120-125. https://doi.org/10.7324/JAPS.2018.8317

4. Justino AB, Miranda NC, Franco RR, Martins MM, da Silva NM, Espindola FS (2018) *Annona muricata* Linn. leaf as a source of antioxidant compounds with *in vitro* antidiabetic and inhibitory potential against *α*-amylase, *α*-glucosidase, lipase, non-enzymatic glycation and lipid peroxidation. Biomed Pharmacother 100:83-92. https://doi.org/10.1016/j.biopha.2018.01.172

5. Liang N, Kitts DD (2014) Antioxidant property of coffee components: assessment of methods that define mechanisms of action. Molecules 19(11):19180-19208. https://doi.org/10.3390/molecules191119180

6. Gutiérrez-Grijalva EP, Antunes-Ricardo M, Acosta-Estrada BA, Gutiérrez-Uribe JA, Heredia JB (2019) Cellular antioxidant activity and in vitro inhibition of *α*-glucosidase, *α*-amylase and pancreatic lipase of oregano polyphenols under simulated gastrointestinal digestion. Food Res Int 116:676-686. https://doi.org/10.1016/j.foodres.2018.08.096

7. Pereira MN, Justino AB, Martins MM, Peixoto LG, Vilela DD, Santos PS, Teixeira TL, da Silva CV, Goulart LR, Pivatto M (2017) Stephalagine, an alkaloid with pancreatic lipase inhibitory activity isolated from the fruit peel of *Annona crassiflora* Mart. Ind Crops Prod 97:324-329. https://doi.org/10.1016/j.indcrop.2016.12.038

8. Balasuriya N, Rupasinghe HV (2012) Antihypertensive properties of flavonoid-rich apple peel extract. Food Chem 135(4):2320-2325. https://doi.org/ 10.1016/j.foodchem.2012.07.023

9. Bhullar KS, Ziaullah Z, Rupasinghe V (2014) *In vitro* regulation of enzymes of the renin-angiotensin-aldosterone system by isoquercitrin, phloridzin and their long chain fatty acid derivatives. Funct Foods Health Dis 4(5):208-221. https://doi.org/ 10.31989/ffhd.v4i5.11

10. Umamaheswari M, AsokKumar K, Somasundaram A, Sivashanmugam T, Subhadradevi V, Ravi TK (2007) Xanthine oxidase inhibitory activity of some Indian medical plants. J Ethnopharmacol 109(3):547-551. https://doi.org/ 10.1016/j.jep.2006.08.020

11. Mohamed NM, Makboul MA, Farag SF, Tarawneh AH, Khan SI, Brooks TA, Wang Y-H, Ross SA (2017) Iridoid and phenylpropanoid glycosides from the roots of *Lantana montevidensis*. Med Chem Res 26(6):1117-1126. https://doi.org/ 10.1007/s00044-017-1817-x

12. Ismail MM, Hassan M, Essam TM (2018) Biological testing and toxicity bioassays in biodegradation: Toward better process control. Toxicity and biodegradation testing, Springer, pp 185-205.

13. Yamamoto K, Miyake H, Kusunoki M, Osaki S (2010) Crystal structures of isomaltase from *Saccharomyces cerevisiae* and in complex with its competitive inhibitor maltose. FEBS J 277(20):4205-4214. https://doi.org/ 10.1111/j.1742-4658.2010.07810.x

14. Akif M, Schwager SL, Anthony CS, Czarny B, Beau F, Dive V, Sturrock ED, Acharya KR (2011) Novel mechanism of inhibition of human angiotensin-I-converting enzyme (ACE) by a highly specific phosphinic tripeptide. Biochem J 436(1):53-59. https://doi.org/ 10.1042/BJ20102123

15. Garcin ED, Arvai AS, Rosenfeld RJ, Kroeger MD, Crane BR, Andersson G, Andrews G, Hamley PJ, Mallinder PR, Nicholls DJ (2008) Anchored plasticity opens doors for selective

inhibitor design in nitric oxide synthase. Nat Chem Biol 4(11):700-707. https://doi.org/10.1038/nchembio.115

16. Pettersen EF, Goddard TD, Huang CC, Couch GS, Greenblatt DM, Meng EC, Ferrin TE (2004) UCSF Chimera—a visualization system for exploratory research and analysis. J Comput Chem 25(13):1605-1612. https://doi.org/ 10.1002/jcc.20084

17. Li H, Robertson AD, Jensen JH (2005) Very fast empirical prediction and rationalization of protein pKa values. Proteins 61(4):704-721. https://doi.org/10.1002/prot.20660

18. Halford B (2014) Reflections on Chemdraw. Chem Eng News 92(33):26-27 https://doi.org/10.1021/cen-09233-scitech1

19. Hospital A, Goñi JR, Orozco M, Gelpí JL (2015) Molecular dynamics simulations: advances and applications. Adv Appl Bioinform Chem 8:37. https://doi.org/10.2147/AABC.S70333

20. Lee T-S, Cerutti DS, Mermelstein D, Lin C, LeGrand S, Giese TJ, Roitberg A, Case DA, Walker RC, York DM (2018) GPU-accelerated molecular dynamics and free energy methods in Amber18: performance enhancements and new features. J Chem Inf Model 58(10):2043-2050. https://doi.org/10.1021/acs.jcim.8b00462

21. Wang J, Wang W, Kollman PA, Case DA (2006) Automatic atom type and bond type perception in molecular mechanical calculations. J Mol Graph Model 25(2):247-260. https://doi.org/10.1016/j.jmgm.2005.12.005

22. Berendsen HJ, Postma Jv, Van Gunsteren WF, DiNola A, Haak JR (1984) Molecular dynamics with coupling to an external bath. J Chem Phys 81(8):3684-3690. https://doi.org/10.1063/1.448118

23. Roe DR, Cheatham III TE (2013) PTRAJ and CPPTRAJ: software for processing and analysis of molecular dynamics trajectory data. J Chemical Theory Comput 9(7):3084-3095. https://doi.org/10.1021/ct400341p

24. Seifert E (2014) OriginPro 9.1: scientific data analysis and graphing software-software review. J Chem Inf Model 54(5):1552. https://doi.org/10.1021/ci500161d

25. Kollman PA, Massova I, Reyes C, Kuhn B, Huo S, Chong L, Lee M, Lee T, Duan Y, Wang W (2000) Calculating structures and free energies of complex molecules: combining molecular mechanics and continuum models. Acc Chem Res 33(12):889-897. https://doi.org/10.1021/ar000033j

26. Ylilauri M, Pentikäinen OT (2013) MMGBSA as a tool to understand the binding affinities of filamin–peptide interactions. J Chem Inf Model 53(10):2626-2633. https://doi.org/10.1021/ci4002475

27. Hayes JM, Archontis G (2012) MM-GB (PB) SA calculations of protein-ligand binding free energies. In: Wang L (ed) Molecular dynamics-studies of synthetic and biological macromolecules, 1st edn. IntechOpen, Croatia, pp171-190. https://doi.org/10.5772/37107

28. Hou T, Wang J, Li Y, Wang W (2011) Assessing the performance of the MM/PBSA and MM/GBSA methods. 1. the accuracy of binding free energy calculations based on molecular dynamics simulations. J Chem Inf Model 51(1):69-82. https://doi.org/ 10.1021/ci100275a

29. Greenidge PA, Kramer C, Mozziconacci J-C, Wolf RM (2013) MM/GBSA binding energy prediction on the PDBbind data set: successes, failures, and directions for further improvement. J Chem Inf Model 53(1):201-209. https://doi.org/10.1021/ci300425v

30. Sitkoff D, Sharp KA, Honig B (1994) Accurate calculation of hydration free energies using macroscopic solvent models. J Phys Chem 98(7):1978-1988. https://doi.org/10.1021/j100058a043
